# miR‐548d‐3p/TP53BP2 axis regulates the proliferation and apoptosis of breast cancer cells

**DOI:** 10.1002/cam4.567

**Published:** 2015-12-13

**Authors:** Qiong Song, Jiangqiang Song, Qimin Wang, Yanling Ma, Nai Sun, Jieyu Ma, Qiu Chen, Guishan Xia, Yanping Huo, Longqiu Yang, Baolin Li

**Affiliations:** ^1^ Department of AnesthesiologyZhengzhou Central Hospital Affiliated to Zhengzhou University195 Tongbai RoadZhengzhouHenan450007China; ^2^ Department of GalactophoreZhengzhou Central Hospital Affiliated to Zhengzhou University195 Tongbai RoadZhengzhouHenan450007China; ^3^ Department of Anesthesiology Huangshi Central Hospita, Affiliated Hospital of Hubei Polytechnic UniversityHuangshi435000China

**Keywords:** Apoptosis, breast cancer, microRNA‐548d, proliferation, TP53BP2

## Abstract

Fast growth and hardly any apoptosis are important characteristics of breast cancer, which assure the spread via invasion and metastasis of breast cancer cells. Inhibition of fast proliferation and induction of apoptosis are critical way to cure this cancer. microRNAs (miRNAs) had been increasingly reported to be the critical regulator of tumorigenesis. In our study, we found that increasing copy number of miR‐548d‐2‐3p is critically involved poor prognosis. We overexpressed miR‐548d‐3p in MDA‐MB‐231cells and found that the proliferation was promoted significantly, whereas the inhibition of miR‐548d‐3p repressed the proliferation of MDA‐MB‐231 cells and also induced the increase in apoptosis. Additionally, we found that miR‐548d‐3p downregulated the expression of TP53BP2 by directly targeting the 3′UTR. We also found that knockdown of TP53BP2 significantly resorted the proliferation and apoptosis regulated by miR‐548d‐3p inhibitor. Our study showed that miR‐548d‐3p/TP53BP2 pathway is critically involved in the proliferation and apoptosis of breast cancer cells and may be new therapeutic target of breast cancer cells.

## Introduction

Breast cancer is a common cancer and causes many deaths of women. To determine the molecular mechanism of the tumorigenesis and development is important for the clinical diagnosis and target therapy. Breast cancer cells have almost unlimited ability of survival with fast proliferation and less apoptosis 1. The proliferation and apoptosis of breast cancer cells has been reported to be regulated by many core transcription factors or chromatin remodeling enzymes which form the circuitries 2, 3, 4, 5. In the core circuitry, TP53BP2 has been reported to be associated with the caner growth 6, 7. It's the critical factor and can cooperate with many other proteins to regulate the tumorigenesis of many kinds of cancer cells. TP53BP2 represses the squamous cell by regulating p63 8. TP53BP2 specifically regulates p53‐dependent apoptosis 9. However, the upregulator of the TP53BP2 and the regulatory mechanism still remains largely unknown.

In addition to protein‐coding genes, microRNAs (miRNAs), about 20 nucleotides‐long noncoding RNAs, have been reported to be the important regulators of repression of mRNA expression and could participate in many kinds of biological processes 10, 11, 12, 13. The cell type‐specific expression signature of miRNAs in cancer cells has been deeply studied for clinical biomarkers and the therapy target in the future. miR‐548d is also critically involved in the regulation of pancreatic cancer 13. miR‐365 has been reported to promote cell proliferation and invasion 14 miR‐29b also is the tumor repressor in breast cancer cells 15, 16, 17. However, characteristic of the miRNA regulatory pathways and the mechanisms which is of great importance to the understanding of breast cancer genesis and development remains largely unknown.

Although the biological activity is realized by proteins which translation can also be repressed by the miRNA, the DNA copy‐number variation (CNV) can also result in the disease including of the cancers 18, 19. Previous studies showed that there was high frequency of CNV of TP53 in serous ovarian cancer 20. In nonsmall cell lung cancer (NSCLC), the DNA copy number of serine/threonine protein kinase1 (AKT1) and AKT2 was upregulated 21. Upregulation of CNV of the cancer related genes, such as Myc, EGFR, ErbB, was associated with the lung cancer 22. These studies suggested that the CNV of genes is the critical related to the cancer, but there still remains largely unknown.

In this study, we found that the upregulating miR‐548d‐3p level of DNA copy number in breast cancer samples in the Cancer Genome Atlas (TCGA). We investigated the roles of miRNA‐548d‐3p in the direct promotion of proliferation and inhibition of apoptosis in breast cancer cells. We then found that the miRNA‐548d‐3p directly targeted the TP53BP2 3′ UTR to downregulate the expression. We also found that overexpression of TP53BP2 repressed the proliferation and promoted the apoptosis, whereas the downregulation of TP53BP2 promoted the proliferation and inhibited the apoptosis. We further determined that the function of miR‐548d‐3p on regulating the proliferation and apoptosis by directly targeting the TP53BP2.

## Material and Methods

### Cell culture

RPMI‐1640 medium (Hyclone, Logan, UT) with 10% fetal bovine serum (FBS) (Gibco, NY) was used to culture breast cancer cell lines at 37°C, 5% CO_2_. Both the culture medium were added 100 U/mL of penicillin sodium (Invitrogen, Life Technology, Carlsbad, CA), and 100 mg/mL of streptomycin sulfate (Invitrogen, Life Technologies).

### Cell proliferation analysis

CellTiter 96^®^ AQueous One Solution Cell Proliferation Assay kit (Promega, Madison, WI) was used to perform the 3‐(4,5‐dimethylthiazol‐2‐yl)‐5‐(3‐carboxymethoxyphenyl)‐2‐(4‐sul‐fophenyl)‐2H‐tetrazolium (MTS) proliferation assay following the instruction. The 490 nm absorption value was detected by microplate reader.

### Cell apoptosis analysis

Cell apoptosis analysis was performed by using AnnexinV‐FITC Cell Apoptosis Detection kit (Sigma‐Aldrich, Shanghai, China).

### Trypan blue staining assay

Trypsin enzyme‐digesting cell were suspended in phosphate buffer saline (PBS). Then added trypan blue solution (0.4%) (Sigma‐Aldrich, Shanghai, China) into it. After 5 min for reaction, used the microscope to calculate the total viable cells (unstained) and total cells (stained and unstained). The cell viability (%) is viable cells/total cells × 100%.

### Knockdown of TP53BP2

We used siRNA‐ TP53BP2‐1 which purchased from Dharmacon (Fairfield, IA) to downregulate the expression of TP53BP2 by reference to the previous study 8. siRNA‐TP53BP2‐1 was purchased from Genepharmer (Shanghai, China) by reference to the previous study 23.

### Overexpression vector of TP53BP2

We generate cDNAs for human TP53BP2 by were inverse transcription from mRNA and amplified by PCR to have the CDS fragment. We inserted the fragment into the pIRES2‐EGFP vector. The primers used to amplify this fragment: PF 5′‐GGCCTCGAGATGCGGTTCGGGTCCAAG‐3′ (xho1 recognition site); RF 5′‐GGCGTCGAC TCAGGCCAAGCTCCTTGTCT‐3′ (sal1 recognition site).

### Construction of luciferase reporter vectors

Fragment of TP53BP2 3′UTR contained the sequence of miRNA binding sites was amplified by PCR from DNA of MDA‐MB‐231 and inserted into the luciferase reporter vector pGL3cM (Promega, Madison, WI). The primers used to amplify this fragment:

PF 5′‐GGCGAGCTCAACTTCCACACAGAATTTTAGTCAA‐3′ (Sal1 recognition site), PR 5′‐GGCTCTTAGAGACATTCTTCATCGCTTTCAA‐3′ (Xba1 recognition site).

TP53BP2‐mutant 3′UTR vector was generated by replacing miRNA seed sequence binding sites from the pGL3cm‐ TP53BP2 3′UTR luciferase reporter vector.

### Luciferase assays

MDA‐MB‐231 cells (5 × 10^5^ cells/per well of 24 wells dish) were co‐transfected with 300 ng of the UTR luciferase reporter, 5 ng Renilla vector, and 25 pmol miRNA‐548d‐3p or control miRNAs (Biotend, Shanghai, China). After 48 h of transfection, the cells were harvested and lysed. The luciferase reporter activity was measured according to the instruction of Dual Luciferase Assay kit (Promega).

### Transfection

Cells growing to about 80% confluence were transfected with the miRNA, siRNA or vectors by using Lipofectamine 2000 (Invitrogen). miR‐548d‐3p inhibitor is the chemically synthesized reverse complementary sequence of the miRNA, which competes with miRNA for target gene mRNAs.

### Western blotting

Cells were lysed in 1 × SDS lysis buffer (Beyotime, Shanghai, China) for electrophoresis. The membrane was incubated by primary TP53BP2 antibody (Abnova, Taiwan, China), GAPDH (sc‐47724; Santa Cruz, Dallas, TX) diluted PBS with 10% FBS. Then the membrane was incubated with secondary antibodies. The results of visualized the signaling was performed by enhanced chemiluminescence (ECL) western blotting substrate (Thermo, Waltham, MA).

### Quantitative real‐time PCR

#### miRNA qRT‐PCR

The total RNA was isolated using RNAiso (Takara, Dalian, China). miRNA was subsequently reverse‐transcribed to cDNA using the miRNA‐specific stem‐loop reverse‐transcription primer (Ribobio, Guangzhou, China). The amount of target gene expression (2^−ΔΔCt^) was normalized via the endogenous small nuclear RNA U6 using miRNA‐specific primers (Ribobio). The reaction conditions were performed according to the instructions from Ribobio Co., Ltd with SYBR Green qPCR Mix (BioRad, Hercules, CA).

#### mRNA qRT‐PCR

The total RNA was isolated using RNAiso (Takara). cDNA was subsequently reverse‐transcribed from mRNA by M‐MLV Reverse Transcriptase (Takara). The PCR included 40 cycles of amplification using the Stratagene Mx3000P system with SYBR Green qPCR Mix (BioRad). Expression of target genes (2^−ΔΔCt^) was normalized against GAPDH. The sequence of primer used in the qRT‐PCR: TP53BP2 PF 5′‐AGCTTGATCGCCTCTATAAGGA‐3′, PR 5′‐CCCTCAGCTCATTAACACGCT‐3′; GAPDH PF 5′‐TGTGGGCATCAATGGATTTGG‐3′, PR 5′‐ACACCATGTATTCCGGGTCAAT‐3′. E‐cadherin PF 5′‐ATTTTTCCCTCGACACCCGAT‐3′, PR 5′‐TCCCAGGCGTAGACCAAGA‐3′. Zeb1 PF: 5′‐CAGCTTGATACCTGTGAATGGG‐3′, PR 5′‐TATCTGTGGTCGTGTGGGACT‐3′. Snail PF 5′‐TCGGAAGCCTAACTACAGCGA‐3′, PR 5′‐AGATGAGCATTGGCAGCGAG‐3′. MMP2 PF 5′‐GATACCCCTTTGACGGTAAGGA‐3′, PR 5′‐CCTTCTCCCAAGGTCCATAGC‐3′.

### Statistical analyses

Statistical significance was determined using a Student's *t*‐test. Values were presented as the mean ± SD. *, **, *** means *P* < 0.05, *P* < 0.01, *P* < 0.001, respectively.

## Results

### High‐level copy number of miR‐548d‐3p is critically involved in the low survival ratio

**Figure 1:**
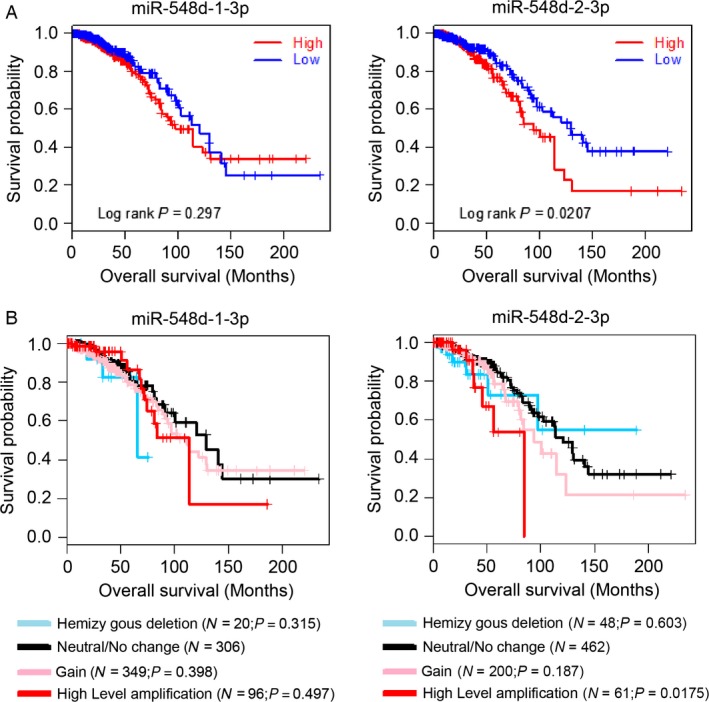
High‐level copy number of miR‐548d‐3p is critically involved in the low survival ratio. (A) Kaplan–Meier (KM) survival curves of breast cancer patients are stratified by their genomic copy‐number value of miR‐548d‐1/3p, miR‐548d‐2/3p. Both survival information and genomic profiles provided by TCGA were used to determine whether the CNV (copy‐number value) show association with overall survival. The data are shown for miR‐548d‐1/3p, miR‐548d‐2/3p. For each miRNA, the breast cancers were classified into high‐ or low‐CNV groups according to whether the log2 value of CNV was greater than their median value. The *P* values from log‐rank tests comparing the two KM curves are shown above each figure and *P* ≤ 0.05 defined by log‐rank test was considered statistically significant. Statistical significance was observed in the curve of miR‐548d‐2/3p (*P* = 0.0207). (B) Kaplan–Meier survival curves for copy‐number alternation clusters derived from GISTIC algorithm. Regions showing statistically significant increase or decrease in genomic copy number was detected using the GISTIC algorithm and were divided into five clusters: Homozygous deletion (blue), Hemizygous deletion (sky blue), Neutral/No change (dark), Gain (pink), and High‐level amplification (red). The data here generated by TCGA was used to determine the affection of copy‐number alternation for overall survival as well. The data are shown for miR‐548d‐1/3p, miR‐548d‐2/3p. For each figure, Log‐rank P value was generated by comparing each two KM curves (Homozygous deletion vs. Neutral/No change, Hemizygous deletion vs. Neutral/No change, Gain vs. Neutral/No change, High‐level amplification vs. Neutral/No change). Statistical significance was observed in the curve of high‐level amplification of miR‐548d‐2/3p (P = 0.0175).

We detected the survival information and genomic profiles provided by the Cancer Genome Atlas (TCGA) about the copy‐number value (CNV) of miR‐548d‐1‐3p(chr8: 123348034‐123348130 [−]) and miR‐548d‐2‐3p(chr17: 67471489‐67471585 [−])which have the same sequence but on the two different genomic DNA showed association with overall survival. Kaplan–Meier (KM) survival curves showed that high level of DNA copy number of miR‐548d‐2‐3p is critical associated with the low survival ratio (Fig. 1A), while the level of miR‐548d‐1‐3p showed no significant association with survival ratio. We further made the GISTIC algorithm analysis to divided the samples into five clusters: Homozygous deletion (blue), Hemizygous deletion (sky blue), Neutral/No change (dark), Gain (pink), and High‐level amplification (red). Then we also determined that the high‐level CNV of miR‐548d‐2‐3p is associated with the low survival ratio (Fig. 1B).

### miR‐548d‐3p plays an important role on regulating the proliferation and apoptosis of MDA‐MB‐231 cells

**Figure 2:**
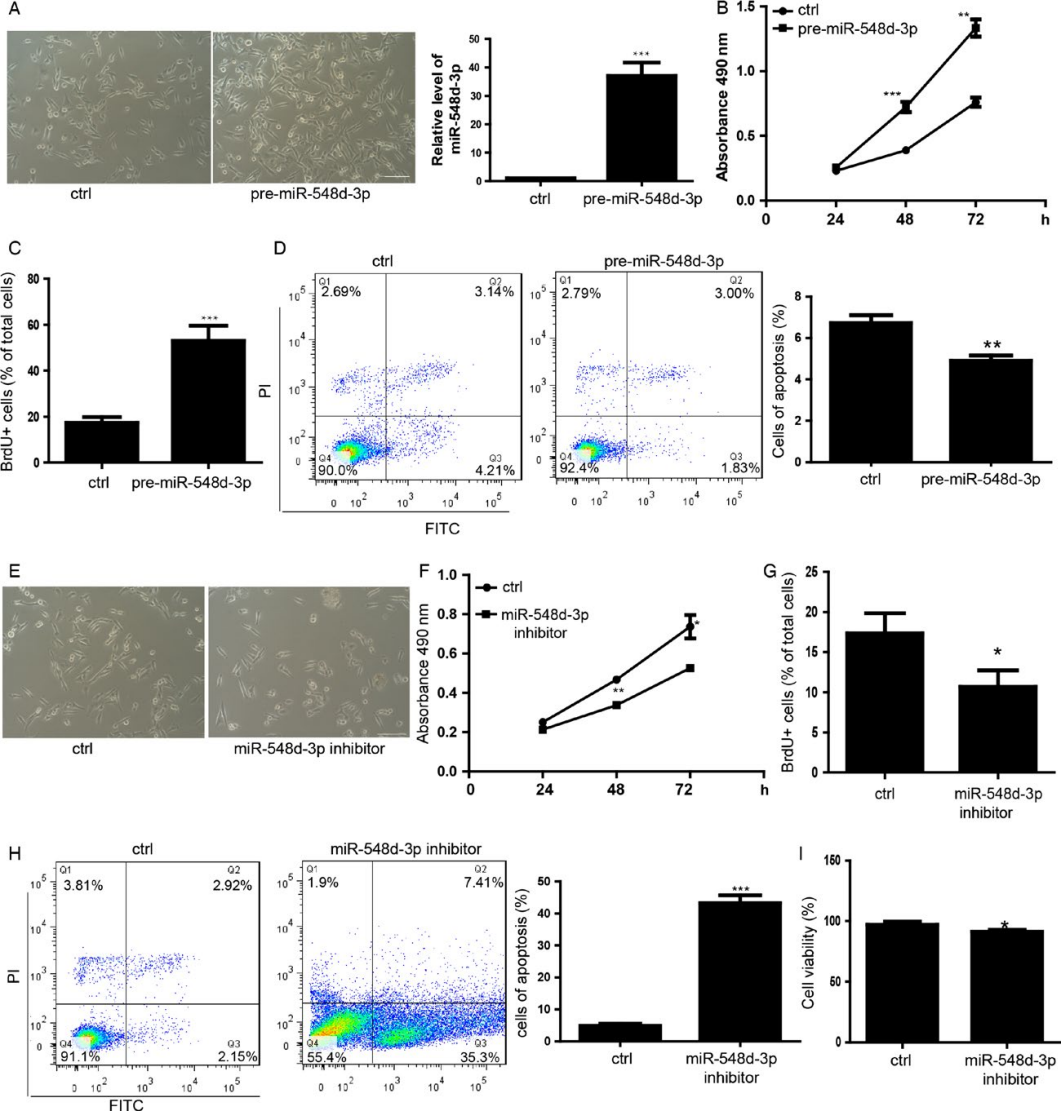
miR‐548d‐3p plays an important role on regulating the proliferation and apoptosis of MDA‐MB‐231 cells. (A) Morphology of MDA‐MB‐231 cells transfected with the pre‐miR‐548d‐3p and control miRNA. The scale bar represents 100 *μ*m. The bottom panel is the effect of transfection of pre‐mir‐548d‐3p in mDA‐MB‐231 cells. Data shown are means ± SD (*n* = 5). ***P < 0.001 versus the corresponding control. (B) MTS assay about the proliferation of MDA‐MB‐231 cells during the 24, 48, 72 h. Data shown are means ± SD (*n* = 3). **P < 0.01,***P < 0.001 versus the corresponding control. (C) Statistics of the BrdU positive cells by BrdU pulse (2 h). *n* = 100 cells, average ± SD, ***P < 0.001. (D) Apoptosis analysis of MDA‐MB‐231 cells. Bottom panel showed the statistics of apoptosis. (E) Morphology of MDA‐MB‐231 cells transfected with the miR‐548d‐3p and control miRNA. (F) The proliferation analysis of MDA‐MB‐231. Data shown are means ± SD (*n* = 3). *P < 0.05,**P < 0.01 versus the corresponding control. (G) Statistics of the BrdU positive cells. *n* = 100 cells, average ± SD, *P < 0.05. (H) Apoptosis analysis of MDA‐MB‐231 cells. Right panel showed the statistics of apoptosis. Data shown are means ± SD (*n* = 3). ***P < 0.01 versus the corresponding control. (I) Statistics of death status of cells treated by sevoflurane or control by trypan staining. Data shown are means ± SD (*n* = 4). *P < 0.05 versus the corresponding control.

In order to detected the function of miR‐548d‐3p, we transfected the miR‐548d‐3p into the MDA‐MB‐231 cells and found that the cells count per field of view is increased by overexpression of miR‐548d‐3p (Fig. 2A). We then performed the MTS proliferation assay to find that miR‐548d‐3p promoted the cell proliferation of MDA‐MB‐231 cells (Fig. 2B). BrdU incorporation assay also showed that miR‐548d‐3p overexpressing MDA‐MB‐231 cells proliferate faster than control group (Fig. 2C). MDA‐MB‐231 cells overexpressed with miR‐548d‐3p showed no significant apoptosis compared with the control group (Fig. 2D). We detected the expression of MET related genes, such as E‐cadherin, Zeb1, Snail and MMP2 by qRT‐PCR and found that there was no significant difference between the cells overexpression with miR‐548‐3p and the control cells (Fig. S1A). In the contrary, miR‐548d‐3p inhibitor which is a chemically synthesized single chain of nuclear acid and has reverse complementary sequence of the miR‐548d‐3p to compete with miRNA for target gene mRNAs could significantly inhibit the cell proliferation (Fig. 2E, F, and G). Additionally, inhibition of miR‐548d‐3p induced the apoptosis of MDA‐MB‐231 cells significantly in MDA‐MB‐231 cells (Fig. 2H). We also found that inhibition of miR‐548d‐3p induced to slight death of MDA‐MB‐231 cells (Fig. 2I).

### miR‐548d‐3p downregulates the expression of TP53BP2 by directly targeting the mRNA 3′UTR

**Figure 3:**
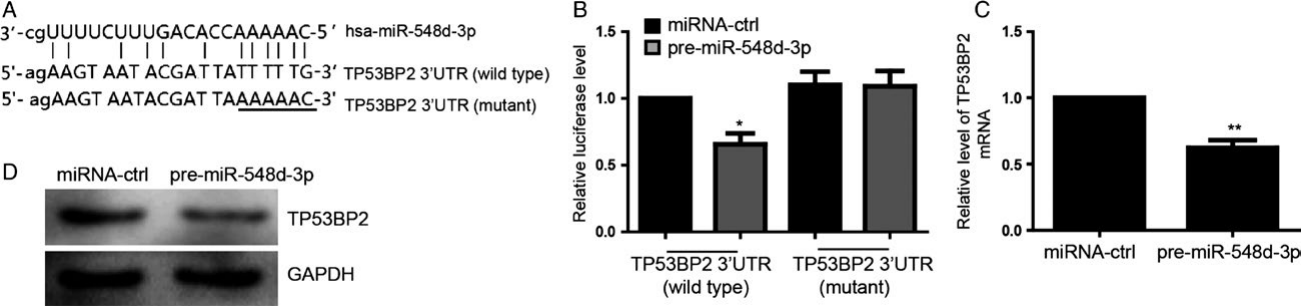
miR‐548d‐3p downregulates the expression of TP53BP2 by directly targeting the mRNA 3′UTR. (A) The TP53BP2 3′UTR reporter in target validation of miR‐548d‐3p. Mutant UTR has a 6 bp replaced by miRNA seed sequence. (B) miR‐548d‐3p specifically represses its targets in the luciferase assay, while cannot influence the mutant UTR luciferase assay. *P < 0.05 versus the corresponding control. (C) mRNA level of TP53BP2 by qRT‐PCR in the MDA‐MB‐231 transfected by pre‐miR‐548d‐3p and control miRNA. **P < 0.01 versus the corresponding control. (D) Protein level of TP53BP2 by western blot.

In order to find the target gene of miR‐548d ‐3p, we made the prediction by using Targetscan, Miranda and found that TP53BP2 is the direct target of miR‐548d‐3p (Fig. 3A). Then we performed the luciferase reporter assay and found that miR‐548d‐3p actually directly targets the wild type of 3′UTR of TP53BP2 mRNA, while the luciferase level of mutant 3′UTR which derived from the wild type 3′UTR luciferase reporter with deletion of the binding sites of miRNA seed sequence showed similar with the control group (Fig. 3B). We further determined that overexpression of miR‐548d‐3p downregulated the expression of endogenous TP53BP2 on both mRNA and protein level in MDA‐MB‐231 cells (Fig. 3C and D).

### TP53BP2 is critically involved in the proliferation and apoptosis of MDA‐MB‐231 cells

**Figure 4:**
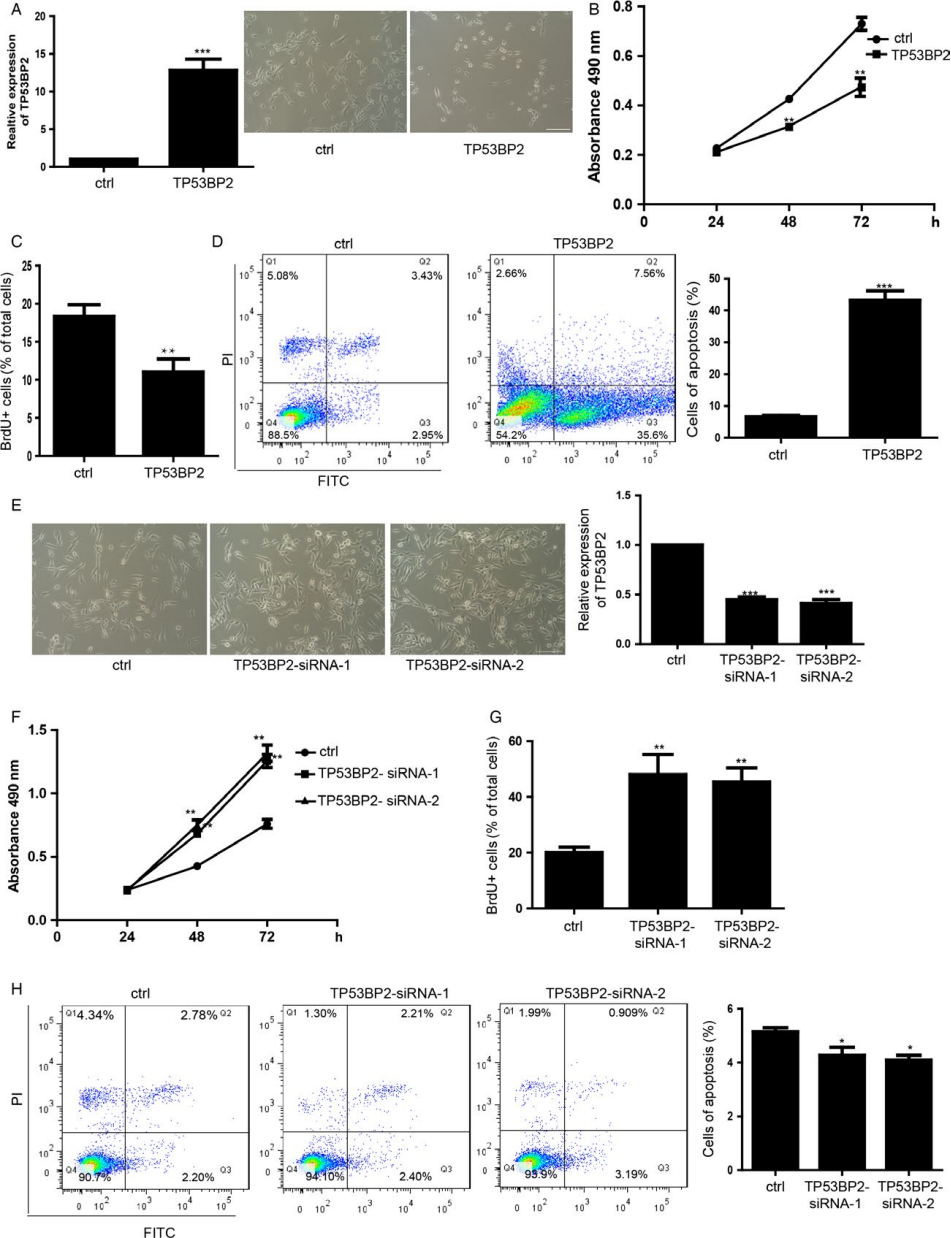
TP53BP2 is critically involved in the proliferation and apoptosis of MDA‐MB‐231 cells. (A) Morphology of MDA‐MB‐231 cells overexpression of TP53BP2 and control vector. The scale bar represents 100 *μ*m. The right panel is the effect of overexpression of TP53BP2 in MDA‐MB‐231 cells. TP53BP2 means the MDA‐MB‐231 overexpressed with TP53BP2. Data shown are means ± SD (*n* = 5). ***P < 0.001 versus the corresponding control. (B) The proliferation analysis by MTS assay. Data shown are means ± SD (*n* = 3). *P < 0.05, **P < 0.01 versus the corresponding control. (C) Statistics of the BrdU positive cells. Data shown are means ± SD (*n* = 100 cells). ***P < 0.01 versus the corresponding control. (D) Apoptosis analysis of MDA‐MB‐231 cells overexpression of TP53BP2 and control vector. Right panel showed the statistics of apoptosis. Data shown are means ± SD (*n* = 5). ***P < 0.01 versus the corresponding control. (E) Morphology of MDA‐MB‐231 cells transfected with the TP53BP2 siRNA. The right penal showed the downregulation of TP53BP2 by siRNA detected by qRT‐PCR. Data shown are means ± SD (*n* = 3). ***P < 0.001 versus the corresponding control. (F) The MTS proliferation analysis of MDA‐MB‐231 during the 24, 36, 72 h. Data shown are means ± SD (*n* = 5). **P < 0.01 versus the corresponding control. (G) Statistics of the BrdU positive cells. Data shown are means ± SD (*n *= 100 cells). **P < 0.01 versus the corresponding control. (H) Apoptosis analysis of MDA‐MB‐231 cells. Right panel showed the statistics of apoptosis. Data shown are means ± SD (*n* = 5). *P < 0.05 versus the corresponding control.

Cells overexpressed with the TP53BP2 showed less cell count than control cells (Fig. 4A). Both of MTS and BrdU incorporation assay showed that overexpression of TP53BP2 repressed the cell proliferation of MDA‐MB‐231 cells (Fig. 4B and C). Additionally, overexpression of TP53BP2 significantly induced the increase in apoptosis compared with the control group (Fig. 4D). In the contrary, downregulation of TP53BP2 by transfecting siRNA increased the cell count (Fig. 4E) and promoted the proliferation of MDA‐MB‐231 cells (Fig. 4F and G). Downregulation of TP53BP2 showed no significant apoptosis in MDA‐MB‐231 cells compared with control group (Fig. 4H).

### miR‐548d‐3p/TP53BP2 axis regulates the proliferation and apoptosis of breast cancer cells

**Figure 5:**
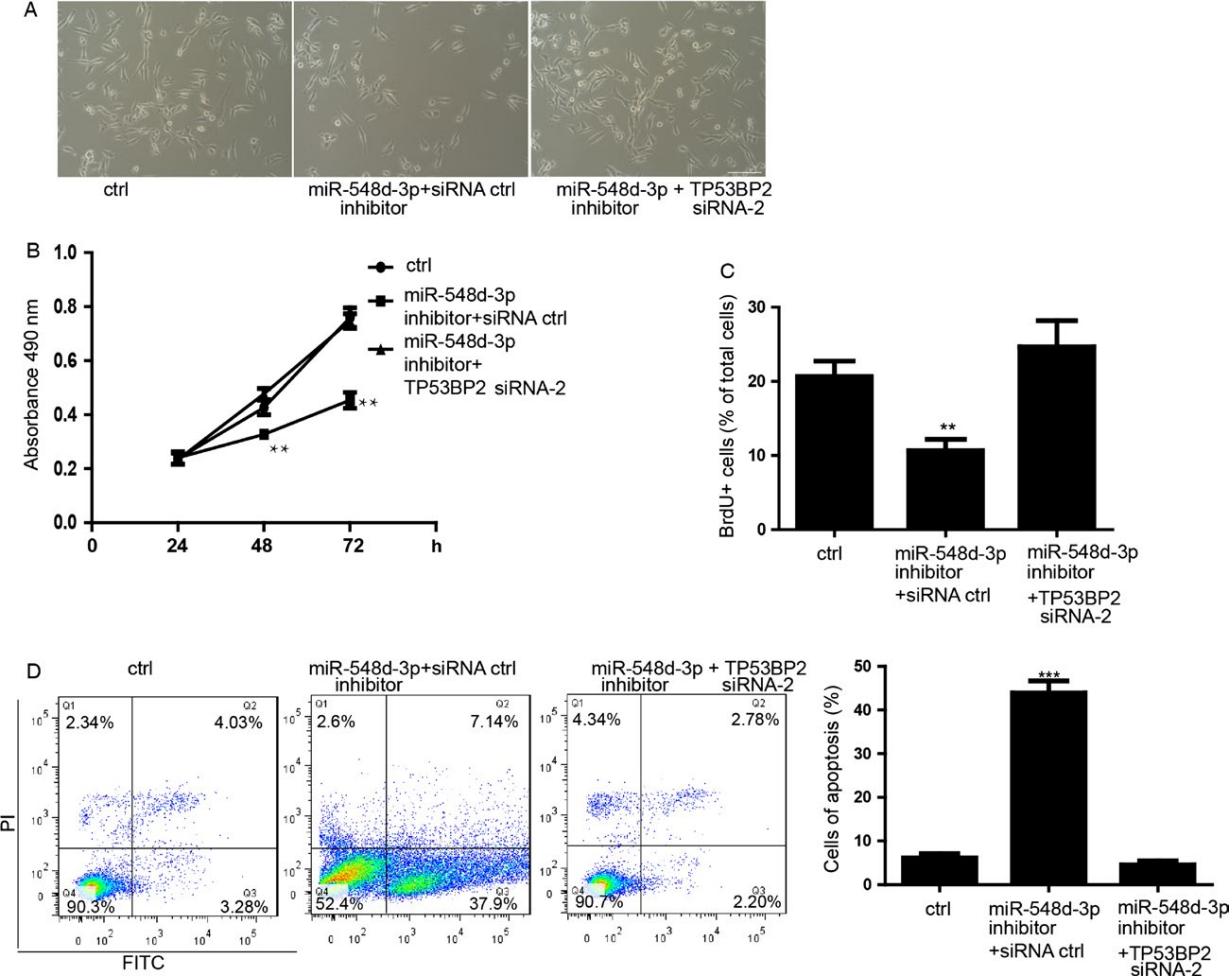
miR‐548d‐3p/TP53BP2 axis regulates the proliferation and apoptosis of breast cancer cells. (A) Morphology of MDA‐MB‐231 cells transfected with imR‐548d‐3p inhibitor plus TP53BP2 siRNA. ctrl means the inhibitor control and siRNA control. (B) The proliferation analysis by MTS assay during the 24, 36, 72 h. Data shown are means ± SD (*n* = 6). *P < 0.05, **P < 0.01 versus the corresponding control. (C) Statistics of the BrdU‐positive cells. Data shown are means ± SD (*n* = 100 cells). **P < 0.01 versus the corresponding control. (D) Apoptosis analysis of MDA‐MB‐231 cells. Right panel showed the statistics of apoptosis. Data shown are means ± SD (*n* = 6). *P < 0.05 versus the corresponding control.

In order to determine whether the function of miR‐548d‐3p is directly via the inhibition of TP53BP2 expression, we performed the rescue experiment. We found that, in contrast to the function of overexpression of miR‐548d‐3p, MDA‐MB‐231 cell with inhibition of the miR‐548d‐3p showed significantly repression of cell count (Fig. 5A). Downregulation of TP53BP2 by siRNA significantly restored the cell count which was less in the MDA‐MB‐231 cells transfected with miR‐548d‐3p inhibitor (Fig. 5A). Additionally, the ability of proliferation of MDA‐MB‐231 cell was restored by downregulation of TP53BP2, which was inhibited by 548d‐3p inhibitor (Fig. 5B and C). Inhibition of miR‐548d‐3p also induced the upregulation of the level of apoptosis (Fig. 5D). We further determined that downregulation of TP53BP2 also blocked the promotion of apoptosis induced by miR‐548d‐3p inhibitor in MDA‐MB‐231 cells (Fig. 5D).

## Discussion

The breast cancer shows fast‐growing incidence in Asian populations, leading cancer‐death among women 24. DNA copy‐number alterations frequently occur in breast cancer patient, which also is defined as the key pathogenetic events. Previous studies showed that mitochondrial DNA copy number is associated with increasing breast cancer risk 25. Germline DNA CNV of *BRCA1/2* is associated with the in familial and early‐onset breast cancer 26. In some breast cancer patients, loss of RAD17, RAD50, and RAP80 s genes which participate into the BRCA1‐dependent DNA repair was associated with significantly increased genomic instability and poor patient survival 27. But the association of CNV of miRNA and the breast cancer still remains largely unknown. In this study, we found that the higher DNA copy number of miR‐548d‐2‐3p which transcripts into the mature miR‐548d‐3p is critical related to the lower survival ratio of breast cancer patients. This result suggested that the upregulation of miR‐548d‐2‐3p genes might associate with the regulation of breast cancer development. The variation of miR‐548d‐1‐3p genes which can also transcript the mature miR‐548d‐3p showed no significant association with the survival ratio, which suggested the more complexity about the regulation of DNA copy‐number alterations of miRNAs. We might further detected that whether the miR‐548d‐3p is the susceptibility genes of breast cancer by using whole‐genome comparative genomic hybridization on microarrays to determine the potentially useful prognostic factor.

Fast proliferation and hardly any apoptosis are the critical characteristics of breast cancer cells. miRNAs have been reported to be the biomarkers of cancer detection and in improving the early diagnosis 28, 29. Additionally, miRNAs are the critical upstream regulators of breast cancer related genes. miR‐10a has been reported to repress the expression of Hox4 to be potential tumor suppressor 30. miRNA‐200c represses the Akt signaling and regulates the expression of E‐cadherin and PTEN, which results the inhibition of adverse drug reactions (ADR) resistance in breast cancer cells 31. Our studies showed that the miR‐548d‐3p significantly promoted the proliferation of breast cancer cells. Downregulation of miR‐548d‐3p significantly increased the apoptosis and repressed the proliferation of breast cancer cells. These results might suggest that the miR‐548d‐3p promoted the development of breast cancer cells. Generally speaking, one miRNA represses many downstream target genes. One gene can be targeted by multiple miRNAs 32. miRNAs and target gene formed the complex regulatory network.

TP53BP2 has been reported to be associated with many kinds of cancers. In gastric cancer TP53BP2 is related to the susceptibility 33. TP53BP2 is a key regulator of epithelial plasticity that connects cell polarity to suppress tumor metastasis 34. ASPP2 cooperates with p53 to repress the tumor growth 35. TP53BP2 can be regulated by STAT1 to form the signaling pathway to suppress tumor 36. These studies showed that the TP53BP2 plays an important role in regulating the tumor genesis. In our study, we found that downregulation of TP53BP2 repressed that proliferation and increased the apoptosis. Whereas the overexpression of TP53BP2 promoted the proliferation and induced less apoptosis of breast cancer cells. These results showed that TP53BP2 significantly repressed the breast cancer generation. But previous study still remains unknown that whether there's miRNA which can directly regulate the TP53BP2. We found that miR‐548d‐3p can directly target the 3′UTR of TP53BP2 and downregulated the expression on both mRNA and protein level. By performing the rescue experiments, we found that downregulation of TP53BP2 can restored the fast proliferation which repressed by the miR‐548d‐3p inhibitor. Additionally, the increasing apoptosis induced by miR‐548d‐3p inhibitor can be restored by downregulation of TP53BP2 in the meantime. These results suggested that the function miR‐548d‐3p on regulating the breast cancer proliferation and apoptosis is directly mediated by TP53BP2. The miR‐548d‐3p/TP53BP2 pathway axis is critically involved in the breast cancer genesis. Our studies also suggested that uncovering the cancer related biomarkers of miRNAs and the miRNAs/mRNAs pathways would be the most important steps in the delineation of breast cancer genesis and further diagnose and treatment.

## Conflict of Interest

None declared.

## Supporting information


**Figure S1A.** Detection of the expression level of MET related genes, such as Snail, MMP2, E‐cadherin, Zeb1 by qRT‐PCR. For all experiments *n* = 3, average ± SD.
