# Supplementary figures and images for: miR‐548d‐3p/TP53BP2 axis regulates the proliferation and apoptosis of breast cancer cells
Source: Cancer Med. 2015 Dec 13;5(2):315–24. doi: 10.1002/cam4.567 (PMC4735782; doi:10.1002/cam4.567)

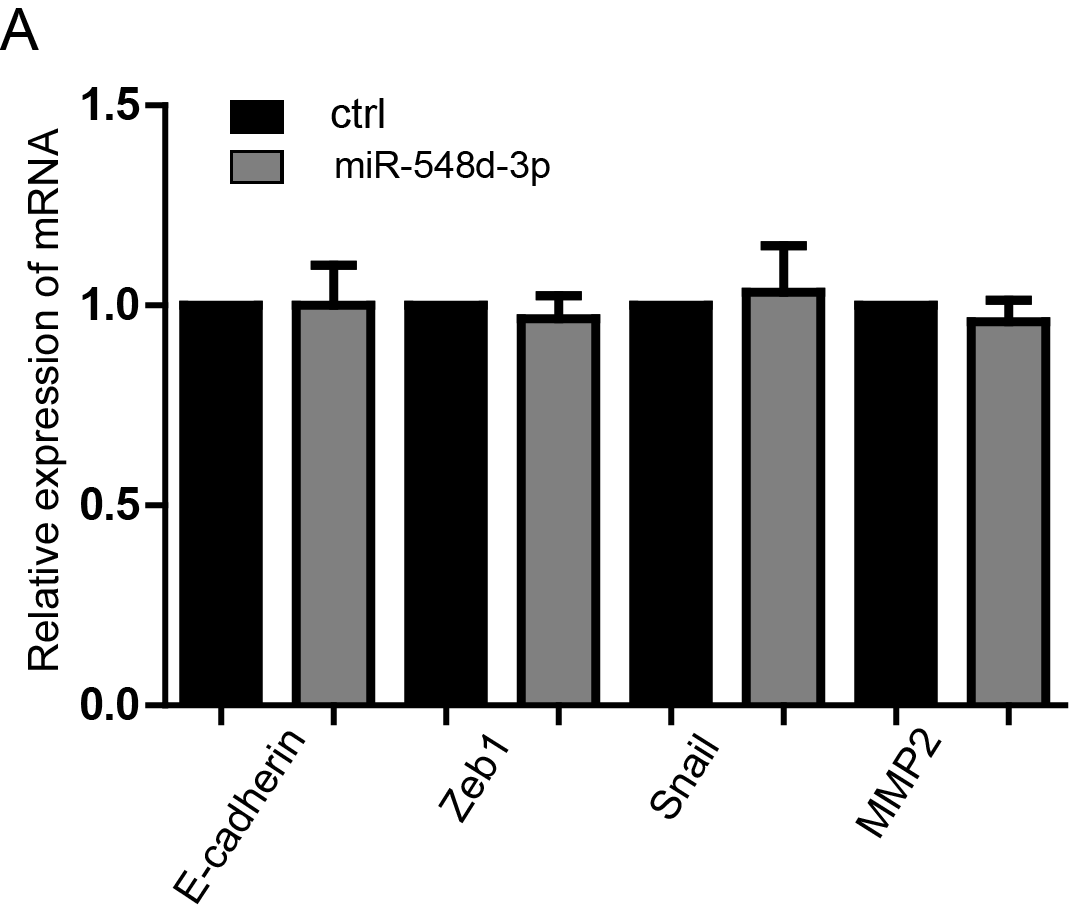

Supplement: Supplementary file 1 — Figure S1A. Detection of the expression level of MET related genes, such as Snail, MMP2, E‐cadherin, Zeb1 by qRT‐PCR. For all experiments n = 3, average ± SD. [file CAM4-5-315-s001.tif]
